# Opportunities to Address Men’s Health During the Perinatal Period — Puerto Rico, 2017

**DOI:** 10.15585/mmwr.mm695152a2

**Published:** 2021-01-01

**Authors:** Beatriz Salvesen von Essen, Katherine Kortsmit, Denise V. D’Angelo, Lee Warner, Ruben A. Smith, Clarissa Simon, Craig F. Garfield, Wanda Hernández Virella, Manuel I. Vargas Bernal

**Affiliations:** ^1^Division of Reproductive Health, National Center for Chronic Disease Prevention and Health Promotion, CDC; ^2^Division of Maternal, Child and Adolescent Health, Puerto Rico Department of Health; ^3^CDC Foundation, Atlanta, Georgia; ^4^Oak Ridge Institute for Science and Education, Oak Ridge, Tennessee; ^5^Department of Pediatrics, Northwestern Medical School, Chicago, Illinois.

Decreased use of health care services (*1*), increased exposure to occupational hazards, and higher rates of substance use (*2*) might contribute to men’s poorer health outcomes when compared with such outcomes for women (*3*). During the transition to fatherhood, paternal health and involvement during pregnancy might have an impact on maternal and infant outcomes (*4*–*6*). To assess men’s health-related behaviors and participation in fatherhood-related activities surrounding pregnancy, the Puerto Rico Department of Health and CDC analyzed data from the paternal survey of the Pregnancy Risk Assessment Monitoring System–Zika Postpartum Emergency Response (PRAMS-ZPER)* study. Fewer than one half (48.3%) of men attended a health care visit for themselves in the 12 months before their newborn’s birth. However, most fathers attended one or more prenatal care visits (87.2%), were present at the birth (83.1%), and helped prepare for the newborn’s arrival (e.g., by preparing the home [92.4%] or purchasing supplies [93.9%]). These findings suggest that opportunities are available for public health messaging directed toward fathers during the perinatal period to increase attention to their own health and health behaviors, and to emphasize the role they can play in supporting their families’ overall health and well-being.

Men are less likely than are women to see or talk to a doctor or other health care professional about their own health (*1*), limiting opportunities for providers to engage with men regarding any health concerns, conditions, or risk behaviors. Men’s higher exposure to occupational hazards (e.g., chemical and physical exposures) and higher prevalence of substance use (e.g., alcohol and illicit drugs) (*2*), compared with women, might contribute to men’s poorer health status (*3*). Men also have a lower life expectancy and higher prevalence of cardiovascular disease and suicide (*2*).

Addressing men’s health needs is especially important during the transition into fatherhood. Associations between the transition into fatherhood and increases in poor physical (*7*) and mental health (*8*) have been observed, both of which might negatively affect men’s involvement in family life (*9*). Paternal involvement during pregnancy has been associated with maternal adoption of healthy prenatal (e.g., early prenatal care and smoking cessation) (*4*) and postpartum behaviors (e.g., breastfeeding) (*5*). Fathers’ engagement with their children has also been associated with improved child outcomes (e.g., cognition, language, social, and emotional development) (*6*).

The PRAMS-ZPER study, a multiphase, collaborative project between the Puerto Rico Department of Health and CDC, was implemented from August 2016 to April 2018 to gather information about experiences related to the prevention and detection of Zika virus infection during pregnancy among women with a recent live birth. To gather information from newborns’ fathers^†^ about their own experiences before and during pregnancy, a survey was implemented during November–December 2017 in hospitals with ≥100 births during 2016. Data from the 30 participating hospitals represented 94% of births in Puerto Rico during the study period. The study sample was identified by randomly selecting newborn delivery dates (clusters) for each hospital. Fathers were initially approached in the hospital shortly after their newborn’s birth. They were eligible to participate if their newborn’s mother had a live birth and was a resident of Puerto Rico. Fathers who consented to participate completed a self-administered survey using paper or electronic forms before the newborn and mother were discharged from the hospital.

PRAMS-ZPER study data were weighted to account for the complex sampling design. Weighted prevalence estimates and 95% confidence intervals (CIs) were calculated for sociodemographic characteristics of participants, their attendance at health care visits during the 12 months before their newborn’s birth, and involvement in selected pregnancy-related activities. Chi-squared tests and 95% CIs were used to assess differences in attendance at health care visits by paternal age, education, employment, and insurance coverage. All analyses were conducted using SAS-callable SUDAAN (version 11.0; RTI International). This study was reviewed and approved by CDC and the University of Puerto Rico Institutional Review Boards.^§^

Among 1,535 eligible men, 1,178 (76.7%) elected to participate. Most were Hispanic (97.8%), aged ≥25 years (74.2%), had some college education or higher (63.8%), were employed (85.1%), and had health insurance (85.9%) (Table 1).

**TABLE 1:** Characteristics of recent fathers and prevalence of reported health care visits during the 12 months before the newborn’s birth, overall and by selected paternal characteristics — Pregnancy Risk Assessment Monitoring System–Zika Postpartum Emergency Response Study, Puerto Rico, 2017

Paternal characteristic	Total		Attendance at any health care visit in the past 12 months		
	Unweighted no.*	Weighted % (95% CI)	Unweighted no.	Weighted % (95% CI)	Chi-squared p-value
**Overall**	1,178	—	1,151	48.3 (45.8–50.7)	—
**Ethnicity**					—^†^
Hispanic	1,132	97.8 (96.9–98.4)	1,106	47.4 (44.9–49.9)	
Non-Hispanic	25	2.2 (1.6–3.1)	25	—^†^	
**Age group, yrs**					0.05
≤24	288	25.8 (23.7–28.0)	282	43.9 (38.9–49.0)	
25–34	566	50.1 (47.7–52.5)	558	48.1 (44.7–51.5)	
≥35	271	24.1 (22.0–26.2)	265	52.1 (47.6–56.6)	
**Education**					<0.01
High school or less	414	36.2 (33.9–38.5)	404	45.5 (41.4–49.6)	
Completed some college or technical school	389	33.5 (31.4–35.7)	385	46.4 (42.3–50.5)	
Completed college or higher	352	30.3 (28.2–32.5)	345	53.9 (49.8–58.0)	
**Employment status**					0.59
Employed	965	85.1 (83.3–86.7)	948	47.9 (45.2–50.5)	
Unemployed	169	14.9 (13.3–16.7)	167	49.7 (43.3–56.2)	
**Insurance**					<0.001
Private	469	40.3 (38.1–42.6)	463	52.9 (49.1–56.7)	
Government insurance/Medicaid	527	45.1 (42.8–47.4)	517	49.3 (45.8–52.8)	
Uninsured	162	14.1 (12.5–16.0)	158	32.7 (26.7–39.3)	
Other	6	0.5 (0.3–0.9)	6	—^†^	

**Abbreviation:** CI = confidence interval.

* Sample size varies because of missing responses in survey.

^†^ <30 respondents, estimate unreliable.

Approximately one half of participants (48.3%) reported attending a health care visit for themselves in the 12 months before the newborn’s birth (Table 1). Attendance at health care visits was higher among men who completed college, compared with men with a high school education or less, and among men who were insured compared with those who were uninsured. Among men attending a health care visit, a regular checkup (60.9%) was the most commonly reported type of visit, followed by dental cleaning (23.4%), and visits for an illness (13.6%) (Figure).

**FIGURE:**
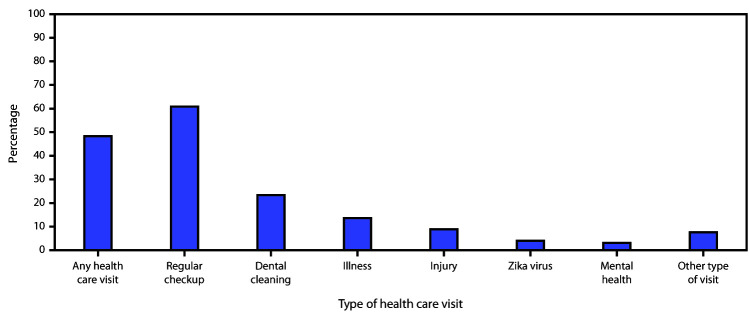
Percentage of health care visits* attended in the 12 months before the newborn’s birth among recent fathers reporting any health care visit, by type of visit^† ^ — Pregnancy Risk Assessment Monitoring System–Zika Postpartum Emergency Response Study, Puerto Rico, 2017 * Among all respondents. ^† ^Among respondents who reported having a health care visit.

Approximately 45.5% of the men were first-time fathers, and 53.1% reported the pregnancy was intended. Most reported living with the newborn’s mother during the entire pregnancy (83.6%); talking with the newborn’s mother about pregnancy, birth, and infant care (91.1%); purchasing supplies such as a crib and stroller (93.9%); preparing the home by setting up a space for the newborn (92.4%); and being satisfied with their level of involvement in the pregnancy (93.3%). Nearly three quarters (71.0%) reported seeking information about pregnancy and birth on the Internet or from other sources (Table 2).

**TABLE 2:** Prevalence of paternal characteristics and participation in selected pregnancy-related activities by recent fathers — Pregnancy Risk Assessment Monitoring System–Zika Postpartum Emergency Response Study, Puerto Rico, 2017

Paternal characteristic	Overall total	
	Unweighted no.*	Weighted % (95% CI)^†^
**Parity**		
First child	515	45.5 (43.1–47.9)
Second child	313	27.4 (25.5–29.4)
Third or later child	308	27.1 (25.0–29.2)
**Pregnancy intention**		
Unintended	418	37.5 (35.3–39.7)
Intended	589	53.1 (51.0–55.3)
Unsure	105	9.4 (8.0–10.9)
**Living with infant's mother during pregnancy**		
Yes, all the time	966	83.6 (81.8–85.2)
Yes, part of the time	109	9.6 (8.3–11.1)
No	79	6.8 (5.7–8.2)
**Pregnancy-related activities**		
**Talked with the newborn’s mother about pregnancy, birth, and caring for a new baby**		
Yes	1,003	91.1 (89.7–92.3)
No	97	8.9 (7.7–10.3)
**Prepared the home by setting up a space for the newborn**		
Yes	1,013	92.4 (91.1–93.4)
No	85	7.6 (6.6–8.9)
**Purchased supplies such as a crib, stroller, clothing, diapers, bottles, or blankets**		
Yes	1,030	93.9 (92.8–94.9)
No	67	6.1 (5.1–7.2)
**Satisfied with pregnancy involvement**		
Yes	1,029	93.3 (92.0–94.4)
No, I wanted to be more involved	68	6.1 (5.0–7.3)
No, I wanted to be less involved	6	0.6 (0.3–1.1)
**Sought information about pregnancy and birth from the Internet/other source**		
Yes	767	71.0 (68.8–73.1)
No	315	29.0 (26.9–31.2)
**Prenatal care visits attendance**		
Attendance at some prenatal care visits	419	36.9 (34.7–39.0)
Attendance at all prenatal care visits	569	50.3 (48.0–52.5)
No	149	12.9 (11.5–14.5)
**Attendance at newborn’s birth**		
Yes	950	83.1 (81.2–84.8)
No	187	16.9 (15.2–18.8)

**Abbreviation:** CI = confidence interval.

* Sample size varies because of missing responses or skip patterns in survey.

^†^ Percentages might not sum to 100% because of rounding.

Overall, 87.2% of men attended prenatal care visits, with 50.3% reporting attending all visits (Table 2). The most common reasons for not attending visits included inability to take time off from work or school (80.6%) and inconvenient appointment times (15.1%) (unpublished data, CDC, 2017). Most men (83.1%) also reported attending their newborn’s birth (Table 2). The most common reasons for nonattendance were that the birth occurred unexpectedly (30.9%) or they were not allowed to attend by medical staff members (26.4%) (unpublished data, CDC, 2017).

## Discussion

Among men in Puerto Rico whose partner had a recent live birth, fewer than one half reported having a health care visit during the 12 months before the newborn’s birth. Despite having limited interaction with the health care system for themselves, approximately 80% of recent fathers in Puerto Rico reported being present during prenatal care visits and at the time of their newborn’s delivery. Approximately 90% of recent fathers reported purchasing supplies for the newborn, and approximately 70% reported seeking information on pregnancy and birth from the Internet or other sources. These findings highlight opportunities for public health messaging directed toward fathers during health care visits throughout the perinatal period. Messaging might also reach new and expectant fathers through other sources or locations they visit frequently around the time of pregnancy, such as pregnancy and infant-related websites and businesses. Public health messaging could focus on increasing men’s attention to their own health and opportunities to help positively influence their family’s overall well-being.

This analysis was strengthened by the large, representative sample of fathers in Puerto Rico reporting on their experiences and behaviors around the time of their partner’s pregnancy and newborn’s birth. The high response rate was comparable to that of the Fragile Families study, a representative father-specific, hospital-based survey in the United States (*10*), and illustrates that fathers are receptive to being approached for such surveys shortly after newborn delivery. This study provides evidence of men’s willingness to participate in pregnancy-related research activities in hospital settings.

The findings in this report are subject to at least four limitations. First, data were collected from men who were present in the hospital with their partner who had a live birth and was a resident of Puerto Rico and might not be representative of other men. Second, survey data were self-reported and thus subject to recall and social desirability bias. Third, data were collected in 2017 shortly after Puerto Rico experienced a Zika virus outbreak and Hurricanes Irma and María when increased stressors might have influenced men’s health care–seeking behaviors and pregnancy involvement. Finally, this analysis did not address health care barriers among recent fathers. The higher proportion of college-educated and insured men who attended health care visits highlights the value of addressing barriers to health care access for less educated and uninsured persons.

The finding of moderate levels of men’s attendance at health care visits for themselves in the 12 months before their newborn’s birth, but high levels of attendance at both prenatal visits and newborn delivery, suggests opportunities for health care providers to engage with expectant and new fathers during perinatal visits. Providers could talk to men about their health and discuss opportunities to positively influence their family’s overall health. In addition, the inclusion of public health messaging targeted toward men through sources for obtaining pregnancy-related information or supplies might help reinforce men’s attention to their health and involvement in pregnancy-related activities. Understanding optimal approaches for integrating health messages for men into activities and encounters during the perinatal period requires additional research.

**Summary**

**What is already known about this topic?**

Men are less likely than are women to seek health care services and are more likely to engage in higher risk health behaviors.

**What is added by this report?**

Fewer than one half (48%) of surveyed recent fathers in Puerto Rico had a health care visit for themselves in the 12 months before their newborn’s birth; however, most attended prenatal care visits with their partner (87%), were present at the birth (83%), and purchased infant supplies (94%).

**What are the implications for public health practice?**

The perinatal period represents an opportunity for public health messaging that encourages men to increase attention to their own health and the role they can play in supporting their families’ overall health and well-being.

## Footnotes

* https://www.cdc.gov/prams/special-projects/zika/index.htm.

† Men were eligible to participate if they were identified by the newborn’s mother as the father or if they were the mother’s current partner. No additional verifications were completed to determine whether the respondent completing the survey was the biological father, the person acknowledging paternity on the birth certificate, or someone else.

§ 45 C.F.R. part 46; 21 C.F.R. part 56.
